# Equine Influenza A(H3N8) Virus Infection in Cats

**DOI:** 10.3201/eid2012.140867

**Published:** 2014-12

**Authors:** Shuo Su, Lifang Wang, Xinliang Fu, Shuyi He, Malin Hong, Pei Zhou, Alexander Lai, Gregory Gray, Shoujun Li

**Affiliations:** South China Agricultural University, Guangzhou, China (S. Su, L. Wang, X. Fu, S. He, M. Hong, P. Zhou, S. Li);; Kentucky State University, Frankfort, Kentucky, USA (A. Lai);; University of Florida, Gainesville, Florida, USA (G. Gray);; Key Laboratory of Comprehensive Prevention and Control for Severe Clinical Animal Diseases of Guangdong Province, Guangzhou (S. Su, M. Hong, P. Zhou, S. Li)

**Keywords:** equine influenza virus, interspecies transmission, contact cohort, cats

## Abstract

Interspecies transmission of equine influenza A(H3N8) virus has resulted in establishment of a canine influenza virus. To determine if something similar could happen with cats, we experimentally infected 14 cats with the equine influenza A(H3N8) virus. All showed clinical signs, shed virus, and transmitted the virus to a contact cohort.

Equine influenza A(H3N8) virus (EIV) remains a major cause of acute respiratory infections in horses (*1*). Epizootics are highly explosive and spread rapidly within and among equine premises. Virus transmission is by direct contact and inhalation. First isolated in 1963, EIV has evolved and diverged into American and Eurasian lineages (*2**,**3*). The American lineage has further diverged into multiple clades: Florida-1 clade predominates in North America and Florida-2 clade in Eurasia (*4*).

In Florida, USA, the etiologic agent of an outbreak of acute respiratory disease among greyhounds in 2004 was identified as EIV. Virologic and serologic analyses indicated that this virus had been circulating among greyhounds for several years before. Serologic evidence of infection was also found for pet dogs (*5*). In Great Britain, retrospective analysis showed that an outbreak of respiratory disease among English foxhounds in 2002 was caused by an EIV (*6*). Likewise, this virus was found to have circulated among greyhounds in the United States before 2004 (*7*). The virus has now been established as canine influenza virus and has spread to other breeds and pet dogs; the virus evolved independently from EIV as a monophyletic lineage (*8*).

During the 2003–2004 outbreak of highly pathogenic avian influenza virus (H5N1) infection in Asia, infections in feline species, including cats, were reported. (*9*). Previously, given the lack of circulating feline influenza virus, feral cats had been believed to be resistant to influenza virus, although an earlier report described susceptibility to A/Hong Kong/68 (H3N2) virus infection and prolonged virus shedding (*10*). Recent serologic and virus isolation studies have shown that cats are susceptible to multiple influenza viruses, e.g., avian-origin canine influenza (H3N2) (*11*), seasonal influenza A(H1N1), and influenza A/H1N1)pdm09 (*12*) viruses. To investigate cats’ susceptibility to EIV infection and virus transmissibility among cats, we conducted an infection and transmission experiment.

## The Study

During 2013–2014, a total of 14 specific pathogen–free domestic shorthair cats, 9–12 weeks of age, were purchased and housed in an accredited Biosafety Level 2 facility at South China Agricultural University, Guangzhou. Results of virus isolation in eggs (nasal and rectal swab samples) and serologic testing determined that these cats were influenza virus free. Experiments were approved by the Institutional Animal Care and Use Committee and monitored by veterinarians. 

The virus used was A/equine/Heilongjiang/SS1/2013, which had been isolated from a mule in northern China (S. Su et al., unpub. data). For virus inoculation, 6 cats were anesthetized with xylazine hydrochloride (30 mg/kg intraperitoneally), after which they were inoculated with virus (10 TCID_50_ [median tissue culture infective dose]) in 1.0 mL of phosphate-buffered saline (0.5 mL in each nostril). One day after inoculation, 5 specific pathogen–free cats (contact cohort) were introduced into the same cages. Three noninfected cats (control cohort) were housed in a different room. Clinical monitoring began 1 day before virus inoculation and continued daily for the next 14 days. Nasal swab samples were collected daily for virus titration in MDCK cells. Serum was collected on postinoculation days 5, 7, 9, 12, and 14 and titrated by hemagglutination-inhibition assay with a 1% horse erythrocyte suspension. On postinoculation day 5, a total of 2 cats from the inoculated group were euthanized by intravenous pentobarbital, and on postinfection day 7, another 2 cats from the inoculated group plus 2 cats from the contact cohort were euthanized. Necropsies were performed, and trachea and lung sections were stained with hematoxylin and eosin and by an immunocytochemistry technique that involved a murine monoclonal antibody specific to EIV hemagglutinin.

**Figure 1:**
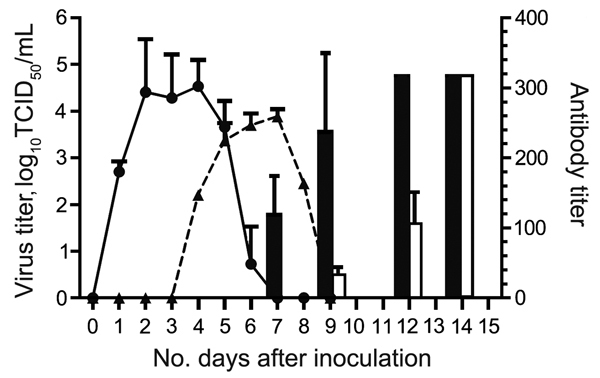
Results of virus titration and hemagglutination-inhibition assay for the cohort of cats inoculated with equine influenza A(H3N8) virus and the contact cohort. Virus shedding was titrated in MDCK cells. Virus titer is shown as log_10_ median tissue culture infective dose (TCID_50_) (solid line and circles, inoculated cohort; dashed line and triangles, contact cohort). Hemagglutination-inhibition assay of serum samples was conducted by using 1% horse erythrocytes (black bars, inoculated; white bars, contact cohort). Error bars indicate SEM.

The cats were susceptible to EIV infection; they showed overt clinical signs, virus shedding, and corresponding histopathologic changes in trachea and lung. Infected cats transmitted the virus to cats in the contact cohort. Overt clinical signs characteristic of acute influenza infection developed in inoculated cats during postinfection days 2–9 (peaking at day 4) and in contact cohort cats during days 4–9 (peaking at day 5); however, average clinical scores were lower for cats in the contact cohort than in the inoculated cohort (Table). Virus shedding was detected for cats in the inoculated group on days 2–5 and in the contact cohort on days 5–6 (Figure 1). This shift of virus shedding correlated with the shift in clinical signs, suggesting that the cohort group was infected by the virus shed from inoculated cats. Likewise, an antibody response was detected for cats in both groups, again 2–3 days later for the contact cohort.

**Table:** Clinical progression for cats in experimental equine influenza A(H3N8) virus inoculation study*

Cohort, cat	No. days after inoculation															
	–1	0	1	2	3	4	5	6	7	8	9	10	11	12	13	14
Inoculated																
A1	0	0	0	2	2.5	3.5	0.5	†								
A2	0	0	0.5	2.5	2.5	3	2	1.5	0.5	0.5	0.5	0	0	0	0	0
A3	0	0	0	2	2.5	2.5	2	0.5	0	†						
A4	0	0	1	2	2	2.5	1.5	0.5	0.5	†						
A5	0	0	0	2	2.5	3	2	†								
A6	0	0	0	2.5	3	3	1	1.5	1.5	0.5	0	0	0	0	0	0
Contact																
B1	0	0	0	0	0	0	2.5	3	2.5	†						
B2	0	0	0	0	0	0	0	0	0	0.5	0	0	0	0	0	0
B3	0	0	0	0	0	1.5	2.5	1.5	1.5	1	1	0	0	0	0	0
B4	0	0	0	0	0	0.5	3	2.5	1.5	†						
B5	0	0	0	0	0	0	0	0.5	0	0	0	0	0	0	0	0
Control																
C1	0	0	0	0	0	0	0	0	0	0	0	0	0	0	0	0
C2	0	0	0	0	0	0	0	0	0	0	0	0	0	0	0	0
C3	0	0	0	0	0	0	0	0	0	0	0	0	0	0	0	0

*Data indicate the sum of clinical scores, as follows: ocular and nasal discharge (0 = no discharge, 0.5 = serous, 1.0 = mild mucopurulent, 2.0 = severe mucopurulent); cough (0 = absent, 0.5 = mild, 1.0 = moderate, 1.5 = persistent, 2.0 = severe with choking or retching); sneezing, dyspnea, and depression (0 = absent, 2 = present); and body temperature (0 = <39.58°C, 2 = ≥39.58°C). Shading indicates days on which some animals had a clinical score >1, showing the shift between the 2 cohorts. †Euthanized on the day indicated.

**Figure 2:**
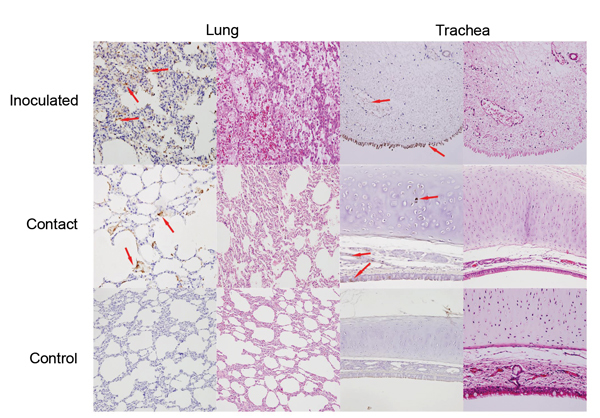
Detection of viral antigens in the respiratory tract of cats inoculated with equine influenza A(H3N8) virus and from a contact cohort. For each tissue type, the left column shows incubation with a monoclonal antibody against equine influenza virus hemagglutinin and the right column shows hematoxylin and eosin staining. Arrows indicate detection of viral antigen (hemagglutinin) expression (brownish staining). Original magnification ×100.

Productive viral infection was evidenced by histopathologic and immunocytochemical examinations. Characteristic lymphocytic infiltration was observed in samples from cats in the inoculated and contact cohorts; intensity was less for cats in the contact cohort (Figure 2). Likewise, EIV antigen was detected in cats in the inoculated and contact cohorts but not in the control cohort. These results indicated productive viral infection in cats in both cohorts. Because specimens were obtained from euthanized animals on the same date (postinfection day 7), the lower intensity of lymphocyte infiltration corresponded to the shift in virus titer (Figure 1) and clinical signs (Table).

## Conclusions 

That cats are susceptible to EIV by direct inoculation is not surprising because infection of cats with various influenza A viruses has been reported. Feline respiratory tract epithelial cells contain sialic acid α-2,3-galactose β-1,3-N-acetyl galactosamine (SA α2,3 gal) receptors for avian and equine influenza viruses and SA α2,6 gal receptors for mammalian influenza virus (*13*). However, our finding of horizontal transmission of EIV among cats is significant. If transmission occurs outside the laboratory, and if the basic reproduction rate is higher than 1.0, then EIV could potentially establish itself and circulate in this new host species. Why it has not yet happened naturally, as it did for canine influenza virus (H3N8), remains to be determined. Possibilities include lower transmission efficiency, lower probability of horse–cat contact, less virus shedding in a laboratory, or feline behavior (less social contact than dogs). 

Because cats in the inoculated and contact cohorts were housed in the same cages, our study could not delineate the route of transmission (direct contact or inhalation). Experiments to elucidate the transmission mechanism are being conducted.

Because we had used a contemporary strain of EIV, to rule out the possibility that the ability to cause clinical infection is unique to this strain, we repeated the experiment with the prototype EIV, A/equine/Miami/63 (H3N8). Although 6 of 6 infected cats showed no overt clinical signs, and virus shedding was not detectable (<1:10), susceptibility was evidenced by seroconversion for 2 of the 6 inoculated cats, although at a low hemagglutination-inhibition titer (1:40 on postinoculation day 14), thereby ruling out the possibility that A/equine/Heilongjiang/SS1/2013 is an aberrant virus. None of the 3 cats in the contact cohort showed clinical signs, shed virus, or had detectable hemagglutination-inhibition titers. This strain-dependent variation in virulence is not unusual for influenza virus. Interspecies transmission of EIV to dogs and establishment of a new lineage of equine influenza virus in dogs were probably a function of that particular EIV strain, as evidenced by finding that so far only 1 EIV-originated canine influenza virus lineage is circulating. However, transmission of EIV to dogs has occasionally occurred, including during the epizootic of EIV in Australia (*14*). Of note, the prototype canine influenza virus is phylogenetically related to the Florida-1 clade of EIV. Whether viruses in this clade have characteristics considered to be “promiscuous” and “plastic” (*15*) remains to be determined. On the basis of our results, we conclude that cats are susceptible to EIV and that the infection can be transmitted by close contact.
